# Normal mode analysis of a relaxation process with Bayesian inference

**DOI:** 10.1080/14686996.2020.1713703

**Published:** 2020-02-10

**Authors:** Itsushi Sakata, Yoshihiro Nagano, Yasuhiko Igarashi, Shin Murata, Kohji Mizoguchi, Ichiro Akai, Masato Okada

**Affiliations:** a Graduate School of Science, The University of Tokyo, Tokyo, Japan; b Graduate School of Frontier Science, The University of Tokyo, Chiba, Japan; c Research and Services Division of Materials Data and Integrated System, National Institute for Material Science (NIMS), Tsukuba, Japan; d Japan Science and Technology Agency, PRESTO, Saitama, Japan; e Graduate School of Science, Osaka Prefecture University, Osaka, Japan; f Institute of Pulsed Power Science, Kumamoto University, Kumamoto, Japan

**Keywords:** Nonlinear optics, data-driven approach, sparse modeling, background estimation, relaxation process, dynamic mode decomposition, Bayesian inference, 404 Materials informatics, Genomics, 204 Optics, Optical applications

## Abstract

Measurements of relaxation processes are essential in many fields, including nonlinear optics. Relaxation processes provide many insights into atomic/molecular structures and the kinetics and mechanisms of chemical reactions. For the analysis of these processes, the extraction of modes that are specific to the phenomenon of interest (normal modes) is unavoidable. In this study we propose a framework to systematically extract normal modes from the viewpoint of model selection with Bayesian inference. Our approach consists of a well-known method called sparsity-promoting dynamic mode decomposition, which decomposes a mixture of damped oscillations, and the Bayesian model selection framework. We numerically verify the performance of our proposed method by using coherent phonon signals of a bismuth polycrystal and virtual data as typical examples of relaxation processes. Our method succeeds in extracting the normal modes even from experimental data with strong backgrounds. Moreover, the selected set of modes is robust to observation noise, and our method can estimate the level of observation noise. From these observations, our method is applicable to normal mode analysis, especially for data with strong backgrounds.

## GRAPHICAL ABSTRACT

**Figure:**
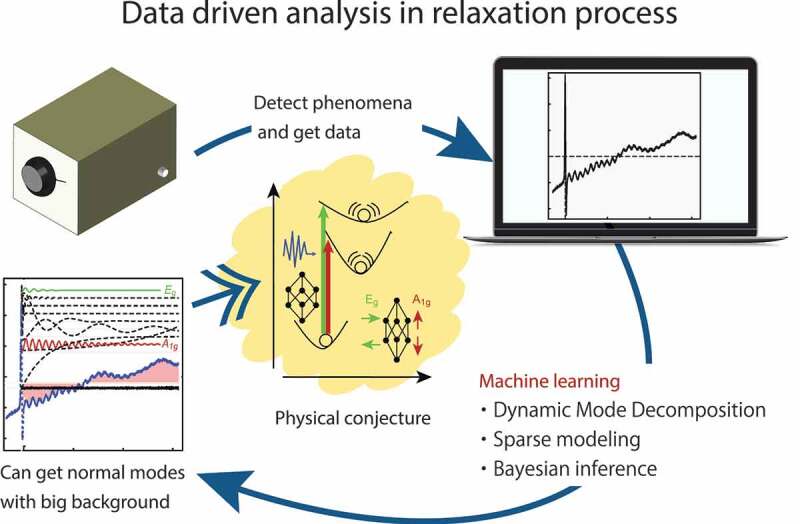

## 1. Introduction

**Figure 1.**
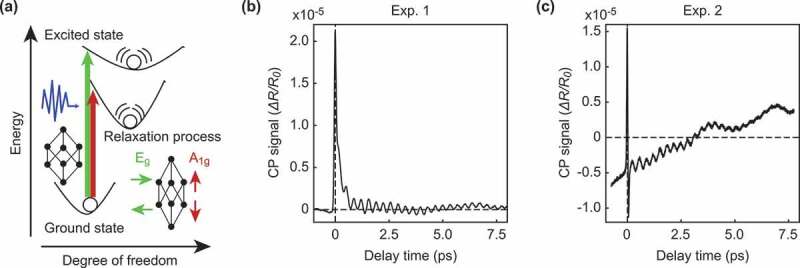
(a) Schematic diagram of coherent lattice vibrations in a (111)-Bi thin film generated by ultrashort optical pulses. In the (111)-Bi thin film, the coherent lattice vibrations corresponding to two different symmetric point groups of $A_{1g}$ and $E_{g}$ are observed by the pump-probe method. (b), (c) Signal data from lattice vibrations in the (111)-Bi thin film. The lattice vibrations start from the initial time 0. The signal contains two damped oscillations, $A_{1g}$ and $E_{g}$. We measured two experimental datasets, Exp. 1 in (b) and Exp. 2 in (c), at different times, so their backgrounds are different, but the signal contains the same damped oscillations.

Relaxation processes are processes by which a perturbed system returns to equilibrium. Measurements of relaxation processes are essential in many fields, such as condensed matter physics [1,2], nonlinear optics [3], structural/reaction chemistry [4], astronomy [5] and atmospheric science [6]. Such processes provide many insights into atomic/molecular structures and the kinetics and mechanisms of chemical reactions. For example, Kim et al. [3] reported evidence of a Mott phase transition by analyzing the structure of a $VO_{2}$ metal crystal from a coherent phonon (CP) signal, which is the relaxation of phonon vibrations. Blagg et al. [7] recently reported the relaxation path and stable structure of the thermal magnetization of a lanthanide compound by analyzing X-ray diffraction patterns. Common to all these areas, the extraction of modes that are specific to the phenomenon of interest (normal modes) from the measured data is unavoidable.

In this study, we focus on the CP signal as a typical case in which the measurement data of a relaxation process include normal modes. Coherent phonons are waves of in-phase atomic vibrations over a macroscopic spatial range, and their signals can be observed by the pomp/probe method [8,9], which uses two ultrashort pulses of light. After the excitation of a substance by the pump pulse, lattice vibrations can be observed as oscillatory changes in optical constants with a delayed probe pulse; this approach has been used to reveal the dynamics of a photoinduced structural phase transition [10–12].

Figure 1(a) shows an example of a (111)-Bi polycrystal as a reference sample [13]. The (111)-Bi polycrystal has the $A_{1g}$ and $E_{g}$ coherent phonon modes. Figure 1(b,c) show typical observations of the CP signal in a (111)-Bi thin film with the same experimental conditions. The difficulty in analyzing the relaxation process represented by the CP signal is that the experimental artifacts and observation noise vary greatly from measurement to measurement. Some signals are relatively easy to analyze owing to the flat background, as shown in Figure 1(b), while others have strong backgrounds and are difficult to analyze, as shown in Figure 1(c). The purpose of CP signal analysis is to extract normal modes from such signals, despite the difficulty. Conventionally, Fourier analysis and wavelet analysis have been employed for CP signal analysis [14–18]. Despite their widespread use, these methods cannot uniquely separate the normal modes since they use trigonometric and wavelet bases, which cannot express damped oscillations.

To address the aforementioned issue, Murata et al. [19] analyzed a CP signal using sparsity-promoting dynamic mode decomposition (SpDMD [20]) based on the fact that CP signals consist of a few damped oscillation modes. SpDMD is a method that decomposes time series data into a sum of damped oscillations and observation noise. The authors isolated candidates of the normal modes from experimental artifacts and observation noise by assuming that the noise was stationary and did not drift over time. Specifically, the authors estimated the amplitude of the noise from the negative time domain (t<0(ps)) in Figure 1(b) since the signal was excited at t=0(ps). However, the negative time domain of CP signals is not necessarily stationary, as shown in Figure 1(c) due to the instability of measurement instruments and lasers. The drift in such backgrounds has a significant influence on the extraction of normal modes.

In this study, we propose a data-driven framework to systematically extract normal modes, even when the background noise is unstable. The isolation of background noise and the extraction of normal modes from measured signals can be recast as a mode selection problem for CP signal estimation. We extend the previously proposed Bayesian LARS-OLS framework [21,22] to be used with SpDMD. Bayesian LARS-OLS is the mode selection framework from the viewpoint of data-driven approach in Bayesian inference. Bayesian LARS-OLS can analytically calculate the performance of a set of SpDMD modes in terms of the Bayesian free energy (FE) [23], which assumes that the measured data can be decomposed into a sum of amplified oscillations and observation noise. We numerically compare the performance of our proposed method with that of the previous method proposed by Murata et al. [19]. Our results consist of the following three points. First, our method succeeds in extracting normal modes from experimental data with strong backgrounds We then use virtual data to examine the effect of noise. The set of selected modes is robust to artificially induced observation noise. Moreover, our proposed method can estimate not only the normal modes but also the level of observation noise. From these observations, our method applies to both mode selection and noise estimation, even for a strong background. These results suggest that our proposed data-driven method is applicable to the relaxation process analysis of material science. Conventionally, a lines of studies conducted Fourier analysis on the relaxation process in material science [1–4]. Our method provides a new way to decompose normal modes by incorporating the physical properties of the relaxation process with Bayesian inference.

## 2. Method

In this section, we introduce a method to extract modes from observation signals of one-dimensional time series, as shown in Figure 1. First, we introduce DMD [24] in Section 2.1. Then, SpDMD [20] is introduced in Section 2.2 as a method that can extract a normal mode expressing the lattice vibrations of a small (sparse) number of modes. Finally, we introduce a framework that extracts the normal mode from the mode extracted by Bayesian LARS-OLS [22] by optimizing the sparse parameter in SpDMD even if the value of experimental noise is unknown in Section 2.3.

### 2.1. Dynamic mode decomposition

DMD is a method of extracting modes with damped or amplified vibrations from data [24–26]. DMD has been utilized in various fields of science and engineering, including fluid mechanics [27], analyses of power systems [28], epidemiology [29], robotic control [30], neuroscience [31], image processing [32], and nonlinear system identification [33].

**Figure 2.**
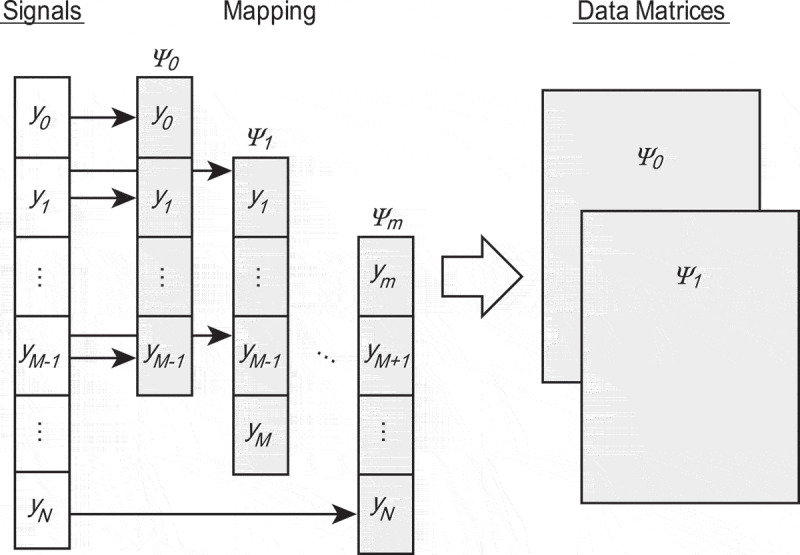
A method of creating a matrix for DMD from the CP signals.

CP signals are measured as one-dimensional time series $\left(y_{0},...,y_{N}\right)$ with a uniform time interval δt, where N is the number of data points. To apply DMD and extract modes from observation signals of one-dimensional time series $\left(y_{0},...,y_{N}\right)$, we map the signals $y_{t}$ to an M-dimensional vector as

(1) $$\psi_{t}=(y_{t},y_{t+1},...,y_{t+M-1}{)}^{T},$$

where the M-dimensional vector is called a state vector. We then obtain m+1 snapshots with a fixed time interval δt, as shown in Figure 2, and define the t-th state vector $\psi_{t}$ as the value at t×δt. Assuming discrete linear dynamics between time t×Δt and (t−1)×Δt, we obtain

(2) $$\psi_{t}=A\psi_{t-1},\;t=\{1,...,m\},$$

where $A\in C^{M\times M}$. The objective is to estimate the matrix A from measured data that describe the discrete linear dynamical system. For this objective, we briefly explain the DMD algorithm according to Jovanović et al. [20]. As shown in Figure 2, we form two data matrices from the snapshot sequence

(3) $$\Psi_{0}=(\psi_{0},...,\psi_{m-1})\in R^{M\times m},$$

(4) $$\Psi_{1}=(\psi_{1},...,\psi_{m})\in R^{M\times m},$$

where m=N−M+1. Using Equation (2), we obtain the following relationship between $\Psi_{0}$ and $\Psi_{1}$:

(5) $$\Psi_{1}=A\Psi_{0}.$$

The data matrix $\Psi_{0}$ is a nonsquare matrix; there is no guarantee of regularity even in the case of a square matrix, so the inverse matrix $\Psi_{0}^{-1}$ does not exist in general. We represent the matrix A that satisfies the above equation by using the Moore-Penrose pseudo-inverse matrix $\Psi^{+}$. The best-fit linear operator A that satisfies Equation 5 is the solution to the following least-squares optimization:

(6) $$A=\underset{A}{arg\;min}{∥\Psi_{1}-A\Psi_{0}∥}_{F}=\Psi_{1}\Psi_{0}^{+}\approx \Psi_{1}V\Sigma^{-1}U^{*}.$$

Here, $U\in R^{M\times r},\Sigma \in R^{r\times r},V\in R^{m\times r}$ are matrices of a singular value decomposition (SVD): $\Psi_{0}=U\Sigma V^{*}$. The eigenvalues and eigenvectors of the matrix A correspond to eigenvalues and eigenmodes of time evolution. The size of the matrix $A\in R^{M\times M}$ is very large. To solve the eigenvalue problem efficiently, instead of the eigenvalue problem of the matrix A, we consider the low rank matrix $\tilde{A}\in R^{r\times r}$ projected onto the singular vectors U. This SVD basis U is called the proper orthogonal decomposition (POD) [34,35].

(7) $$\tilde{A}=U^{*}AU=U^{*}\Psi_{1}V\Sigma^{-1},$$

Next, we computed the eigendecomposition of $\tilde{A}$:

(8) $$\tilde{A}\Phi =\Phi D_{\mu }.$$

The diagonal matrix $D_{\mu }=diag(\mu_{1},\mu_{2},..,\mu_{r})$ means the eigenvalues of $\tilde{A}$, which are same in A. The corresponding eigenvectors of A is represented the follows:

(9) Φ=UW,

where $W\in C^{r\times r}$ is a matrix of $\tilde{A}$’s eigenvectors. Corresponding to DMD eigenvalues μ, we called the columns $\{\phi_{1},\phi_{2},...,\phi_{r},\}\;\phi \in C^{M}$ as DMD eigenmodes. Applying Equation (2) repeatedly, the state vector $\psi_{t}$ at step t from the initial vector $\psi_{0}$ can be represented as

(10) $$\psi_{t}=A^{t}\psi_{0}\approx \left(\Phi D_{\mu }{\Phi^{†}}^{t}\right)\psi_{0}=\sum_{i=1}^{r}\alpha_{i}\phi_{i}\mu_{i}^{t}.$$

Here, we define $\alpha ={\left(\alpha_{1},\alpha_{2},\ldots ,\alpha_{r}\right)}^{T}\equiv \Phi^{†}\psi_{0}\in C^{r}$ as the mode amplitude, which corresponds to the coefficients of each mode. The vector corresponding to the eigenvalue is $\mu ={\left(\mu_{1},\mu_{2},\ldots ,\mu_{r}\right)}^{T}\in C^{r}$.

We need to estimate α from the data, which is the solution to an optimization problem. From the above expressions, the data matrix $\Psi_{0}$ can be represented as

(11) $$\Psi_{0}\approx \Phi D_{\alpha }V_{and}.$$

Here, we define $D_{\alpha }=diag\left(\alpha_{1},\alpha_{2},..,\alpha_{r}\right)$ and a r×m Vandermonde matrix representing the latent structure in the temporal direction

(12) $$V_{and}=\left(\begin{array}{cccc} 1 & \mu_{1} & \ldots & \mu_{1}^{m-1}\\ 1 & \mu_{2} & \ldots & \mu_{2}^{m-1}\\ \vdots & \vdots & \ddots & \vdots \\ 1 & \mu_{r} & \ldots & \mu_{r}^{m-1} \end{array}\right)\in C^{r\times m}.$$

The coefficient α of each mode is expressed using the pseudo-inverse matrix $\Phi^{+}$ of the dynamic mode Φ. Here, we obtain α by solving the least squares problem.

(13) $$\alpha_{LS}=\underset{\alpha }{arg\;min}J(\alpha ),$$

(14) $$J(\alpha )\equiv {∥\Psi_{0}-\Phi D_{\alpha }V_{and}∥}_{Fro}^{2},$$

${∥X∥}_{Fro}$ represents the Frobenius norm of the matrix X. J(α) is the objective function that needs to be minimized to obtain the mode amplitude α. In Appendix A, we present the correspondence between the DMD modes and the relaxation process.

### 2.2. Sparse estimation of DMD

We assume prior knowledge that the normal modes representing the lattice oscillations of a substance among modes extracted by DMD are few (sparse). We introduce SpDMD [20] to obtain the sparse solution of the mode amplitude. SpDMD is a combination of DMD and the least absolute shrinkage and selection operator (Lasso) [36], which automatically extracts a small number of components for the purpose of explaining data. We solve the following $\ell_{1}$-regularized optimization problem.

(15) $$\alpha_{Sp}(\gamma )=\underset{\alpha }{arg\;min}\;J(\alpha )+\gamma \sum_{i=1}^{r}|\alpha_{i}|,$$

Here, γ represents the weight of the $\ell_{1}$ regularization term. If γ takes a significant value, α becomes a more sparse solution. If SpDMD gives the regularization parameter γ, we obtain a unique mode set.

### 2.3. Mode selection by Bayesian LARS-OLS

We deal with mode selection in Bayesian inference by considering the prior of α. Although we optimized α with $\ell_{1}$ regularization as shown in above, the $\ell_{1}$ regularization develops a bias of the basis coefficient. In this section, we present a well-known technique for preventing the bias, called ‘polishing’ [37], within the framework of Bayesian inference. Such framework is known as Bayesian LARS-OLS [22] and we extend this framework to the mode selection problem in SpDMD. Bayesian LARS-OLS framework uses Bayesian free energy [23] to evaluate the goodness of model fit. We can determine the optimal sparsity γ and select the set of DMD modes based on the criteria. In Section 2.3.1, we first introduce the ‘polishing’ method. Next, in Section 2.3.2, we introduce the Bayesian LARS-OLS and Bayesian free energy (FE).

#### 2.3.1. Polishing

We regress the mode amplitude α again in terms of the candidate modes selected by SpDMD to prevent the bias of the coefficient. The technique is called ‘polishing’ [36]. We deal with the ‘polishing’ in mathematics and introduce the prior of α condition in Bayesian inference by considering parameter c(γ). We define an indicator c(γ) representing whether the modes is selected or not by SpDMD:

(16) $$c(\gamma )\equiv \left\{i|\alpha_{i}\gamma \neq 0\right\},$$

where i is the index of the modes. In other words, the indicator c(γ) is the set of indices of which DMD mode was estimated as nonzero. We solve the constrained optimization problem of Equation (17) using the set c(γ) with suffix i depending on the $\ell_{1}$ regularization term γ in SpDMD.

(17) $$\begin{array}{rl} & \underset{\alpha }{minimize}\;\;\;J(\alpha )\\ & subject\;to\;\;\;\alpha_{i}=0,\forall i\notin c(\gamma ) \end{array}$$

This equation means that we minimize J(α) under the constraint $\alpha_{i}=0$, and α depends on γ only through c.

#### 2.3.2. Evaluation of decomposed modes

In this section, we deal with the aforementioned ‘polishing’ method in a Bayesian inference. The ‘polishing’ has two different purposes: mode search by $\ell_{1}$ regularization and regression. Least angle regression and ordinary least squares (LARS-OLS) [37] is a framework of regression analysis that performs basis search and regression in a stepwise manner. Igarashi et al. considered the LARS-OLS as Bayesian inference (called Bayesian LARS-OLS) and proposed a method that achieves the basis search in the Bayesian free energy criterion [21,22]. We extend their method to be able to apply the material’s dynamics such as a relaxation process. In Bayesian LARS-OLS framework, we consider DMD as a problem of linear regression and express it by a probability model.

The model of DMD as described above can be written as $\hat{y}=X^{*}\alpha$ where $X^{*}$ is a matrix determined from the eigenvalues μ and eigenvectors ψ of DMD. Please see Appendix B for the detail derivation. The optimization of $\ell_{2}$ loss (Equation 14) for above linear model corresponds to the maximum likelihood estimation under the assumption that the observation noise is the independent and identically distributed (i.i.d.) Gaussian. Overall, we can write the DMD estimator as follows:

(18) $$y=X^{*}\alpha +\epsilon .$$

When we estimate the modes X and sparsity γ as $\hat{X}$ and $\hat{\gamma }$, we define a combination of modes as $\hat{c}=c(\hat{\gamma })$. We note that c(⋅) computes $\hat{c}$ through SpDMD by using $\hat{X}$ and $\hat{\gamma }$. To evaluate $\hat{c}$, we consider a posterior distribution $P\left(\hat{c}|y\right)$. From the Bayesian theorem, the posterior is

(19) $$P(\hat{c}|y)=\frac{P(y|\hat{c})P(\hat{c})}{P(y)}.$$

Here, P(y) is constant. We define the prior distribution $P(\hat{c})$ to be uniform and use the following relationship:

$$P(\hat{c}|y)\propto P(y|\hat{c})$$

(20) $$=\int P(y|\alpha ,X,\hat{c}\;)P(\alpha |X,\hat{c}\;)P(X|\hat{c}\;)d\alpha dX.$$

Based on the above assumption (Equation 18), the following holds: $P(y|\alpha ,X,\hat{c})∼N(X^{*}\alpha ,\sigma^{2})$. We assume that the matrix X, which characterizes modes, does not depend on the combination $\hat{c}$, so we define

(21) $$P\left(X|\hat{c}\right)=\delta (\hat{X}).$$

This equation means that the combination of modes does not change either the eigenvalue of the time evolution μ or the dynamic mode ϕ, where $\delta \left(\cdot \right)$ is the Dirac delta function. Substituting Equation (21) into Equation (20), we obtain

(22) $$P(y|\hat{c})=\int P\left(y|\alpha ,\hat{X},\hat{c}\right)P\left(\alpha |\hat{X},\hat{c}\right)d\alpha .$$

Here, the $P\left(\alpha |\hat{X},\hat{c}\right)$ is a prior probability and the $P\left(y|\alpha ,\hat{X}\right)$ is a distribution of data generation. The $P(y|\hat{c})$, called the marginal likelihood, represents the likelihood of the indicator $\hat{c}$ estimated from the data. The negative marginal log-likelihood,

(23) $$FE(\hat{c})=-\log P(y|\hat{c}),$$

is often referred to as the Bayesian free energy (FE) [23]. The FE criterion evaluates the goodness of model fit. In our criteria, the indicator c, which means whether the DMD mode was selected or not, determines the models and FE. The indicator c with the smallest FE represents the best basis set. To calculate FE analytically, we define the prior distribution $P\left(\alpha |\hat{X},\hat{c}\right)$ as follows in the cases of $\alpha_{i}=0$ and $\alpha_{i}\neq 0$.

(24) $$P(\alpha_{i}|\hat{X},i\in \hat{c})=CN(0,\sigma_{pri}^{2})$$

$$=\frac{1}{\pi \sigma_{pri}^{2}}\exp\left(-\frac{1}{\sigma_{pri}^{2}}\alpha_{i}^{*}\alpha_{i}\right),$$

(25) $$P(\alpha_{i}|\hat{X},i\notin \hat{c})=\delta (\alpha_{i})=\delta (0).$$

Here, the coefficient α is complex number, we define $P(\alpha |\hat{X},\hat{c})$ as a complex Gaussian distribution [38]. From the obtained FE $(\hat{c})$, we estimate the variance $v=\sigma^{2}$ of the noise and the variance $v_{pri}=\sigma_{pri}^{2}$ of the prior distribution as values that minimize the FE (c). By repeatedly solving the self-consistent equations until convergence, the values of v and $v_{pri}$ that minimize the FE $(\hat{c})$ are obtained. In this way, we can analytically calculate the FE. The reason why we can analytically calculate the FE $(\hat{c})$ is that the parameter X of the DMD’s eigenvalues and eigenvectors has already been determined.

## 3. Results and discussion

We focus on experimental signals of a bismuth (Bi) thin film as typical examples of relaxation processes. We deposited a Bi thin film on a sapphire substrate with a thickness of 150 nm and used a reflective pump-probe spectroscopy method [16] to obtain the CP signals. The signal background varied from measurement to measurement due to experimental instabilities. We analyzed two typical signals. One signal has a background close to steady-state, as shown in Figure 1(b), and the other signal has a strong background, as shown in Figure 1(c). In Sections 3.1 and 3.2, we explain the advantage of our proposed method. Finally, in Section 3.3, by using virtual data, we describe the robustness to noise and the noise estimation performance.

**Figure 3.**
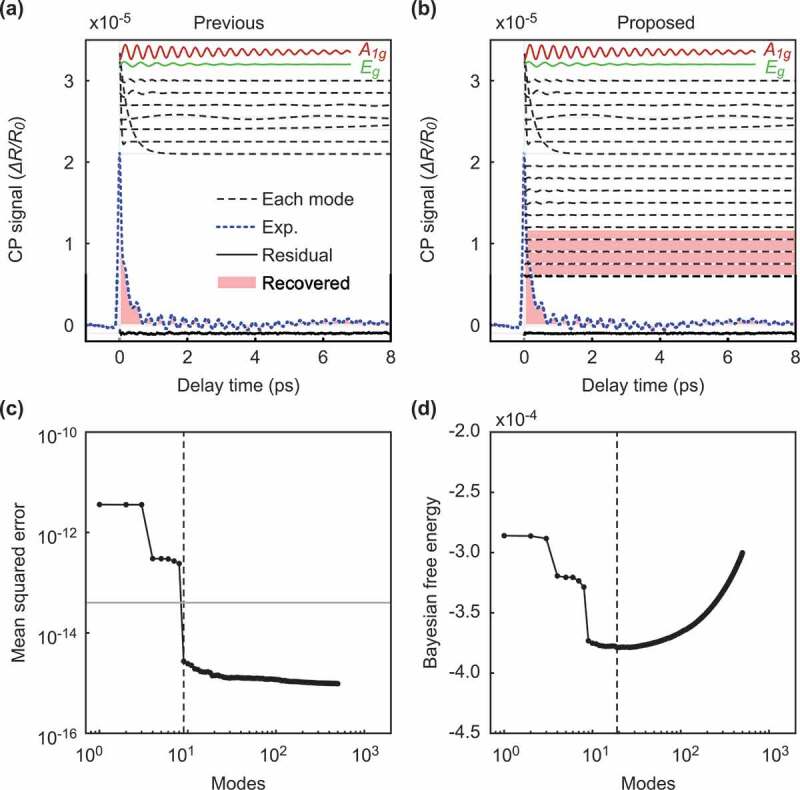
Comparison of our method and the previous method for data with a weak background. The figures on the left side show the result of the previous method, and the figures on the right side show the result of our method. (a-b): The intensity of the signals according to the delay time. The black dotted and solid lines show each selected mode and the residual signal between the original signal and the reconstructed signal, respectively. Among the selected modes, the normal modes $A_{1g}$ and $E_{g}$ are shown in red and green, respectively. The solid blue line shows the original experimental signal, and the pink filled area is the recovered signals with the selected mode. (c-d): The value of the metric used for the mode selection according to the number of modes. The previous method uses mean squared errors (MSEs) as the metric (c), and our method uses the Bayesian FE (d). The vertical dotted lines represent the point of the optimal number of modes selected by each method. The horizontal gray line in (c) shows the estimated variance of the noise.

### 3.1. Background close to steady-state

**Figure 4.**
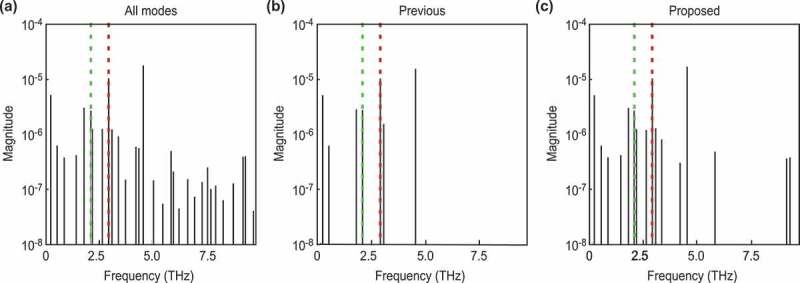
Frequencies of each decomposed mode for data with a weak background. The vertical axes represent the absolute values of the mode amplitudes, and the horizontal axes represent the mode frequencies. The red lines correspond to the frequency of $A_{1g}$, and the green lines correspond to the frequency of $E_{g}$. (a): All decomposed modes. (b): The modes selected by the previous method for the estimated noise. (c): The modes selected by our method for the Bayesian FE.

We analyzed the weak-background data in Figure 1(b). The experimental data had N = 2433 data points from t = 0.00 to 8.00 ps at an interval of Δt = 8/2433 ps. The data matrices $\Psi_{0}$ and $\Psi_{1}$ were generated according to Figure 2 with M = 1000. We prepared the weight of the sparsity term γ in Equation (15) as 600 units with equal intervals on a log scale between $\gamma =10^{-9}$ and $10^{-2}$.

Figure 3(a) shows the reconstruction of the original signal with the optimal modes selected by the previous method. As shown by the red and green lines in Figure 3(a), the previously proposed method successfully extracted the normal vibration modes $A_{1g}$ and $E_{g}$. Moreover, the other selected modes are denoted by the black dotted lines and correspond to a weak background in the experimental data [19]. By using the selected modes, the recovered signals coincide with the experimental data, as shown in the pink filled area and the blue line of Figure 3(a). The residual signal between the original signal and the reconstructed signal is close to zero, as shown by the solid bottom lines of Figure 3(a). In Murata et al. [19], assuming prior knowledge that the normal modes representing lattice oscillations of the substance of interest were sparse, the authors selected the modes extracted by DMD as explained in Section 2.1. The previous method determined the optimal number of modes to be nine, where the curve of the MSE curve crosses the estimated noise variance $\sigma_{est}^{2}=4.00\times 10^{-14}$, as shown in Figure 3(c). Note that the authors estimated the noise variance from the variance of the negative time signal [19]. On the other hand, the proposed method also succeeded in extracting the normal modes from the experimental data without using speculative noise information, as shown in Figure 3(b). In the proposed method, we estimated the noise variance in objective data using Bayesian inference and optimized the number of normal modes as 19 by minimizing the Bayesian FE, as shown in Figure 3(d). Our proposed method can obtain an estimate of the observed noise level from the Bayesian FE. We estimated that the standard deviation of the noise was $\sigma_{FE}=3.56\times 10^{-8}$, while the value of the noise estimated in the previous study was $\sigma_{est}=2.00\times 10^{-7}$.

To qualitatively investigate the selected normal modes, we compare the results of a frequency spectrum analysis of the selected normal modes with the results of a previous study [16]. Hase et al. [16] reported that the two normal modes of (111)-Bi, $A_{1g}$ and $E_{g}$, have frequencies of 2.91 and 2.07 THz, respectively. Figure 4 quantitatively shows the frequency of each mode, where the horizontal axis represents the frequency calculated from the eigenvalue of the extraction mode, and the vertical axis represents the absolute value of the mode amplitude. Figure 4(b,c) show the modes selected by the previous method [19] and the proposed method, respectively, where both methods assumed that the original signal consisted of a few modes. The figures show that both methods successfully extracted the normal modes at 2.90 and 2.09 THz. These modes correspond well to $A_{1g}$ (2.91 THz) and $E_{g}$ (2.07 THz), respectively.

**Figure 5.**
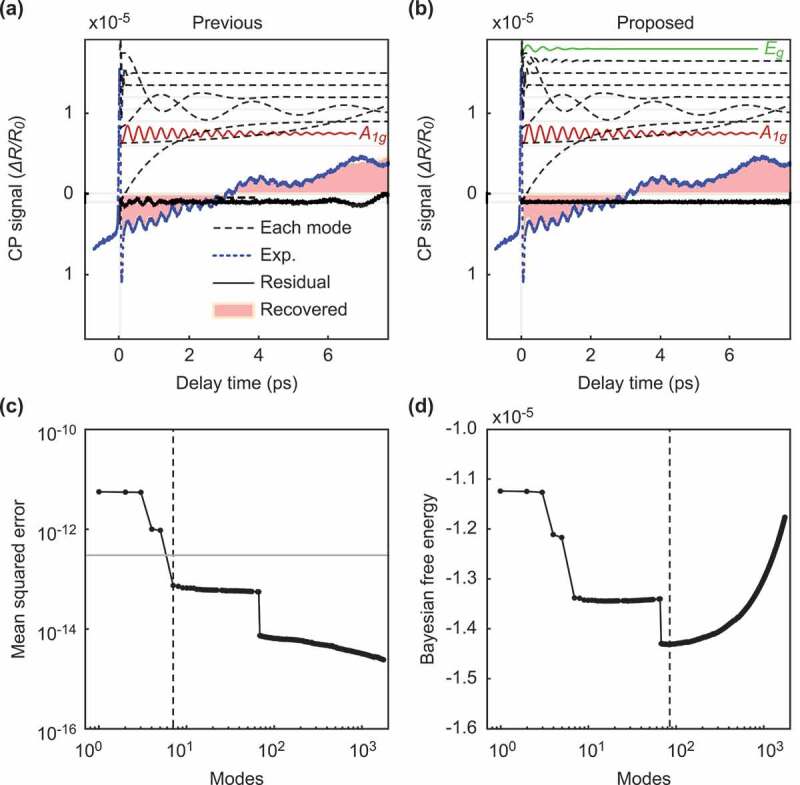
Comparison of our method and the previous method for data with a strong background. The figures on the left side show the result of the previous method, and the figures on the right side show the result of our method. (a-b): The intensity of the signals according to the delay time. (c-d): The value of the metric used for the mode selection according to the number of modes. The meaning of each color and line style is the same as in Fig. 3.

### 3.2. Strong background

**Figure 6.**
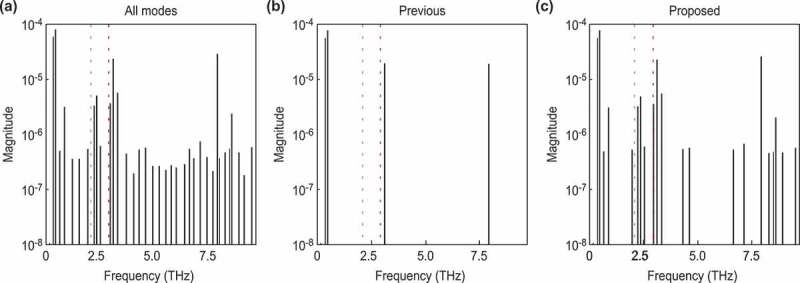
Frequencies of each decomposed mode for data with a strong background. The meaning of each color and line style conforms to that in. Figure 4

In contrast to the previous section, the assumption regarding the noise estimation by Murata et al. [19] does not hold when the data have a strong background, which can cause the normal mode selection to fail. Let us consider the strong-background case. The experimental data had N = 9740 data points from t = 0.00 to 7.50 ps at an interval of Δt = 7.5/9740 ps. The data matrices $\Psi_{0}$ and $\Psi_{1}$ were generated according to Figure 2 with M=4970. We prepared the weight of the sparsity term γ in Equation (15) as 600 units with equal intervals on a log scale between $\gamma =10^{-7}$ and $10^{-2}$.

Figures 5 and 6 show the results for a strong background. The previous method determined seven modes as shown in Figure 5(c). Although the selected modes included the $A_{1g}$ mode, they did not include the $E_{g}$ mode. On the other hand, our method selected 85 modes, as shown in Figure 5(d), which successfully included both the $A_{1g}$ and $E_{g}$ modes. Our method also has an advantage in terms of the residuals, as shown by the solid black line in Figure 5(a,b). The residual signal extracted by our method is steady, whereas the previous method includes a vibration component in the residual signal.

As in Figure 4, we performed a frequency spectrum analysis of each mode to quantitatively evaluate the difference from the normal modes obtained in a previous study [16] and to ensure that normal modes can be extracted quantitatively (Figure 6). As in Figure 4(a–c), Figure 6(a) shows the modes extracted from DMD, and Figure 6(b,c) show the modes selected by the previous method [19] and the proposed method, respectively. Figure 6(b) shows that the previous method successfully extracted the mode at 2.96 THz, which corresponds to the $A_{1g}$ (2.91 THz) mode band; however, it failed to extract the $E_{g}$ (2.07 THz) mode band. On the other hand, Figure 6(c) shows that the proposed method successfully extracted the normal modes at 2.96, and 2.34 THz. These modes correspond to the $A_{1g}$ (2.91 THz) and the $E_{g}$ (2.07 THz) modes bands, respectively. The estimated errors were 0.05 and 0.27 THz, respectively. This latter error seemed to be influenced by the intense fluctuating background components. To decompose such phonon modes, which have a weak amplitude and short decay time, we should improve the experimental conditions to reduce such experimental artifacts.

Let us consider the noise estimation results. We estimated that the standard deviation of the noise was $\sigma_{FE}=8.08\times 10^{-8}$, while the value of the noise estimated in the previous research was $\sigma_{est}=5.33\times 10^{-7}$. In both the strong- and weak-background data, $\sigma_{est}$ is approximately six times larger than $\sigma_{FE}$. The error of the noise estimation has a critical effect on the normal mode selection in the data with a strong background. The previous method failed to select the $E_{g}$ mode, because the noise level estimated by the previous method was larger than the noise level estimated by the proposed method. Because the amplitude of the $E_{g}$ mode was smaller than the $A_{1g}$ mode, it was considered that the $E_{g}$ mode deviated from the selected mode. However, since our method estimates noise without a stationary assumption for an experimental artifact, we succeeded in selecting normal modes with small amplitudes. Our method is effective, especially for data with a strong background. By applying our method to CP analysis, we can select normal modes from measurement signals without considering the experimental situation. Since the normal modes were known for (111)-Bi, it was possible to find normal modes from candidates of selected modes obtained by mode selection.

### 3.3. Virtual CP data

**Figure 7.**
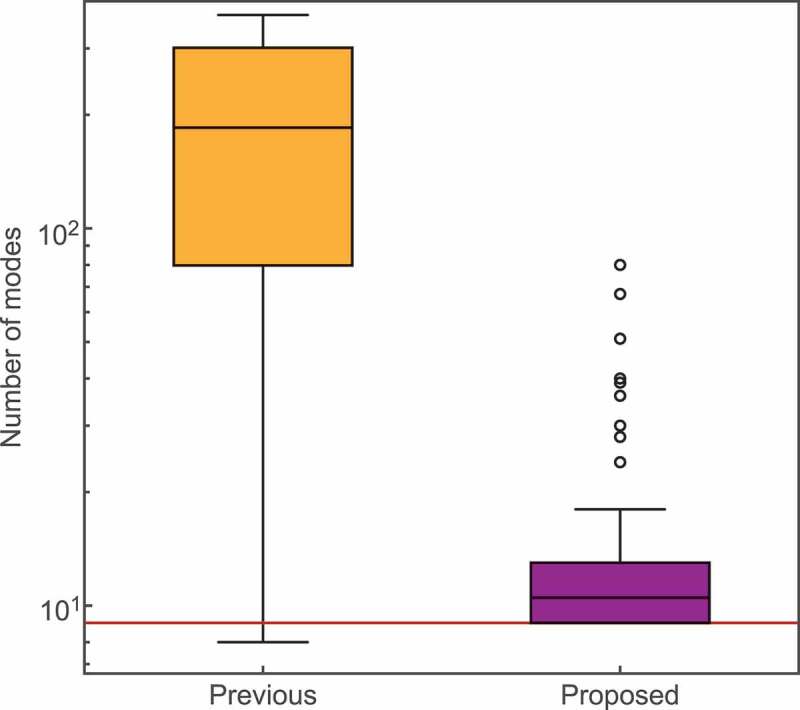
Results of the mode selection of 100 virtual CP signals. The box plots show the number of selected modes for the previous method (left) and the proposed method (right). The horizontal red line shows the correct number of modes: nine.

Finally, we investigate the performance of the previous method and the proposed method against virtual CP data to evaluate the effect of noise. Let us consider the results of the virtual CP data. We created virtual data by injecting Gaussian noise ($\sigma ∼8.0\times 10^{-8}$) into the reconstructed signal from nine modes chosen by the previous method in the weak-background (Figure 3(a)). We prepared 100 virtual datasets with different samples of noise.

Figure 7 shows the number of selected modes. We plotted the distributions of counts as a box plot. The previous method selected 191±114 modes, while our method selected 14±11 modes. We note that the number of modes selected by proposed method was always greater than the correct number of modes of nine. Since there is no negative time domain in the generated virtual data, the noise level used to determine the mode in the previous method was the generated noise level ($\sigma ∼8.0\times 10^{-8}$). In the result of the previous method, the selected numbers varied considerably according to the noise, and the mode number tended to be much larger than the correct number of modes. On the other hand, our method has minimal dispersion and can select a number that is closer to the correct mode number from noisy data. These results show that our method is much more robust to noise than the previous method.

Let us consider the noise estimation results. The estimated noise level by the FE was $\sigma ∼9.4\pm 1.5\times 10^{-8}$. Since the true noise level is $\sigma ∼8.0\times 10^{-8}$, our method can estimate the amplitude of the original noise from an observed signal whose noise value is unknown. This result indicates that it is possible to quantitatively evaluate the amplitude of the noise without using a heuristic technique.

## 4. Conclusions

In this study, we extended Bayesian LARS-OLS to be used in SpDMD for the selection of normal modes in a relaxation process. We numerically compared the performance of our proposed method to the previous method proposed by Murata et al. [19]. The following three results showed the effectiveness of the proposed method based on data of the CP signal of a (111)-Bi thin film. First, our method succeeded in extracting a small number of modes, including the normal modes, from experimental data even with significant background trends. Then, we used virtual data to examine the effect of noise. The selected set of modes was more robust to observation noise than the conventional method. Finally, our proposed method allowed to estimate not only normal modes but also the level of observation noise quantitatively. From these observations, our proposed method is applicable to normal mode analysis of relaxation processes, especially for data with strong backgrounds, which broadens the applicability of data-driven approach in analyzing of relaxation phenomena in material science.

## Appendix A. Correspondence between the DMD basis and the form of damped oscillations

This chapter explains how the form of the damped oscillations representing the relaxation process can be expressed using the DMD basis. The time evolution of the DMD basis is $V_{and}$ in the (12) expression Here, when we converted each eigenvalue $\mu_{j}$ of DMD into $\mu_{j}=r_{j}\exp(i\theta_{j})$ in the complex polar coordinate system, each element $\mu_{j}^{n}$of $V_{and}$ becomes $\mu_{j}^{n}=\exp\;(n\ln r_{j})\;\exp\;(in\theta_{j})$.

Assuming that the initial time of the time series data is $t_{0}$ and that the time interval Δt is constant, the relationship between the time step n and the time t is $n=t-t_{0}/\Delta t$. Now, $\mu_{j}^{n}$ is expressed using t as follows

(A1) $$\mu_{j}^{n}=R_{j}\exp\left(\frac{\ln r_{j}}{\Delta t}t\right)\exp\left(i\frac{\theta_{j}}{\Delta t}t\right).$$

where $R_{j}$ is $R_{j}=\mu_{j}^{-t_{0}/\Delta t}$ We rewrite this equation in the form of damped oscillation as follows

(A2) $$\mu_{j}^{n}=R_{j}\exp\left(-\frac{t}{\tau_{j}}t\right)(\cos2\pi f_{j}t+i\sin2\pi f_{j}t).$$

Then, $\tau_{j}$ and $f_{j}$ are

(A3) $$\tau_{j}=-\frac{\Delta t}{\ln r_{j}},\;f_{j}=\frac{\theta_{j}}{2\pi \Delta t}.$$

$\tau_{j}$ is the damping time constant and $f_{j}$ is the frequency. Thus, the (10) formula can be rewritten in terms of time evolution from a discrete form to a continuous form as follows

(A4) $$\psi_{n}=\sum_{j=1}^{r}R_{j}\exp\left(-\frac{t}{\tau_{j}}\right)(\cos2\pi f_{j}t+i\sin2\pi f_{j}t),$$

where $R_{j}$ is $\alpha_{j}R_{j}\phi_{j}$Eq. (A4) contains a term for damping where $f_{j}=0$ and a term for vibration where $f_{j}\neq 0$. Each decomposed form is as follows

(A5) $$\psi_{n}=\sum_{\rho }R_{\rho }\exp\left(-\frac{t}{\tau_{\rho }}\right)+$$

$$\sum_{\nu }R_{\nu }\exp\left(-\frac{t}{\tau_{\nu }}\right)(\cos2\pi f_{\nu }t+i\sin2\pi f_{\nu }t),$$

where $\sum_{\rho }$ is the sum of elements for which $f_{j}=0$, and $\sum_{\nu }$ is the sum of elements for which $f_{j}\neq 0$. y(t) is observation data and contains only real components as follows.

(A6) $$\psi_{t}=\sum_{\rho }C_{\rho }\exp\left(-\frac{t}{\tau_{\rho }}\right)+$$

$$\sum_{\nu }\exp\left(-\frac{t}{\tau_{\nu }}\right)(a_{\nu }\cos2\pi f_{\nu }t+b_{\nu }\sin2\pi f_{\nu }t),$$

where $C_{\rho }=R_{\rho },\;\;a_{\nu }=2\;Re(R_{\nu }),\;\;b_{\nu }=-2\;Im(R_{\nu })$. Since only the real component is obtained by discarding the pure imaginary component, the degree of freedom of the complex conjugate remains. As a result, the vibration mode of $f_{j}\neq 0$ is obtained by two modes of the same value in the space of only real numbers.

## Appendix B. Transformation to original signal shape

In this chapter, we explain the operation to restore the data matrix obtained by SpDMD to the original one-dimensional time series signal. From the result of (1), the original signal data $y=y_{0},...,{y_{N}}^{T}$ can be expressed as follows

(B1) $$2y=\left(\begin{array}{c} y_{0}\\ y_{1}\\ \vdots \\ y_{M-1}\\ y_{M}\\ y_{M+1}\\ \vdots \\ y_{N} \end{array}\right)+\left(\begin{array}{c} y_{0}\\ y_{1}\\ \vdots \\ y_{N-M}\\ y_{N-M+1}\\ y_{N-M+2}\\ \vdots \\ y_{N} \end{array}\right)=\left(\begin{array}{c} \psi_{0}\\ {{\chi^{\prime }}_{M}}^{T} \end{array}\right)+\left(\begin{array}{c} {\chi_{0}}^{T}\\ \psi_{N-M+1} \end{array}\right).$$

Here, $\chi ,\chi^{\prime }$ is a column vector which is a pair of state vectors ψ of DMD introduced for the reconstruction of the original time series and can be expressed using

(B2) $$V_{and}^{\prime }=\left(\begin{array}{cccc} \mu_{1} & \mu_{1}^{2} & \cdots & \mu_{1}^{N-M+1}\\ \mu_{2} & \mu_{2}^{2} & \cdots & \mu_{2}^{N-M+1}\\ \vdots & \vdots & \ddots & \vdots \\ \mu_{r} & \mu_{r}^{2} & \cdots & \mu_{r}^{N-M+1} \end{array}\right)\in C^{r\times N-M+1},$$

(B3) $$T_{i}=\left(\begin{array}{cccc} \Phi_{i,1} & 0 & \cdots & 0\\ 0 & \Phi_{i,2} & \cdots & 0\\ \vdots & \vdots & \ddots & \vdots \\ 0 & 0 & \cdots & \Phi_{i,r} \end{array}\right)\in C^{r\times r},\;$$

and $\alpha ={\left(\alpha_{1},\alpha_{2},...,\alpha_{r}\right)}^{T}$ as

(B4) {\chi _i} = \sum\limits_{j = 1}^r {\alpha _j}{{\rm ⁋hi} _{ij}}{v_j} = {{\boldsymbol \alpha} ^{\rm{T}}}{{{\bf T_i}}{{\boldsymbol V}_{{\rm{and}}}} \in {{\mathbb C}^{1 \times N-M+ 1}},

(B5) $$\chi_{i}^{\prime }=\sum_{j=1}^{r}\alpha_{j}\Phi_{ij}v_{j}^{\prime }=\alpha^{T}T_{i}V_{and}^{\prime }\in C^{1\times N-M+1}.$$

Therefore, it becomes ${\chi_{0}}^{T}=V_{and}^{T}T_{0}\alpha ,\;{{\chi^{\prime }}_{M}}^{T}={V^{\prime }}_{and}^{T}T_{M}\alpha$. The next thing to do is to display matrix with $y=X^{*}\alpha$. By using

(B6) $$*D_{\mu }^{N-M+1}=\left(\begin{array}{cccc} \mu_{1}^{N-M+1} & 0 & \cdots & 0\\ 0 & \mu_{2}^{N-M+1} & \cdots & 0\\ \vdots & \vdots & \ddots & \vdots \\ 0 & 0 & \cdots & \mu_{r}^{N-M+1} \end{array}\right),\;$$

$\psi_{0}$ corresponds to $\Phi \alpha \in C^{M}$, $*\psi_{N}$ corresponds to $\Phi D_{\mu }^{N-M+1}\alpha \in C^{M}$. Eventually, it becomes

(B7) $$y=\frac{1}{2}\left(\begin{array}{c} \Phi \\ {V^{\prime }}_{and}^{T}T_{M} \end{array}\right)\;\alpha +\frac{1}{2}\left(\begin{array}{c} V_{and}^{T}T_{0}\\ \Phi D_{\mu }^{N-M+1} \end{array}\right)\;\alpha =X^{*}\alpha .$$

Here, we note

(B8) $$X^{*}=\frac{1}{2}\left(\begin{array}{c} \Phi \\ {V^{\prime }}_{and}^{T}T_{M} \end{array}\right)+\frac{1}{2}\left(\begin{array}{c} V_{and}^{T}T_{0}\\ \Phi D_{\mu }^{N-M+1} \end{array}\right).$$
